# P-306. Characterization of a Rare Hospital-Associated MRSA Lineage

**DOI:** 10.1093/ofid/ofae631.509

**Published:** 2025-01-29

**Authors:** Emily Felton, Sarah Kennedy, Jessica Jackson, Ariana Virgillio, Eleonora Cella, Deanna Becker, Amorce Lima, Suzane Silbert, Taj Azarian, Kami Kim, Lindsey Shaw

**Affiliations:** University of South Florida, Tampa, Florida; University of South Florida, Tampa, Florida; University of South Florida, Tampa, Florida; USF Morsani College of Medicine, Tampa, Florida; University of Central Florida, Orlanda, Florida; Tampa General Hospital, Tampa, Florida; Tampa General Hospital, Tampa, Florida; Tampa General Hospital, Tampa, Florida; University of Central Florida, Orlanda, Florida; University of South Florida, Tampa, Florida; University of South Florida, Tampa, Florida

## Abstract

**Background:**

The adaptive power of *Staphylococcus aureus* enables it to cause a wide variety of infections and readily develop resistance to antibiotics. It does this through extensive variability in its genomic repertoire, not only via laterally acquired elements but also through core genome evolution.

**Methods:**

Herein we use whole genome sequencing to study the genomic epidemiology of *S. aureus* strains circulating within Tampa General Hospital (TGH).

**Results:**

While characterizing the genetics of mupirocin resistance, we identified a rare ST belonging to hospital-associated clonal complex 5. The elevated rate of mupirocin resistance in this ST, ST3390 (83.2% total, high-level resistance 55.5% and low-level resistance 27.7%), was particularly striking. To date, there have only been 6 recorded instances of this lineage globally, whilst this work identified 36 individual strains from TGH alone. Genome analysis of our strains identified numerous completely conserved non-synonymous mutations in genes relating to DNA damage response, metabolism, adhesion/biofilm formation, iron acquisition and peptidoglycan biosynthesis compared to CC5 isolates. Additionally, exploration of AMR genes detected the presence of a unique dual SCCmec type, with two-thirds of strains possessing components of both SCCmecIa and SCCmecIIa. Phenotypically, all ST3390 strains lack the characteristic staphyloxanthin pigment that gives *S. aureus* its name. Additionally, ST3390 isolates display high levels of cytotoxicity towards human neutrophils compared to other CC5 strains.

**Conclusion:**

Collectively, this work provides a deeper understanding of the genetic variability of *S. aureus* in clinical settings, leading to a better comprehension of in-hospital bacterial population dynamics and the evolution and spread of contemporary isolates.

**Disclosures:**

**All Authors**: No reported disclosures

